# Effects of Attentional Focus on Motor Performance and Physiology in a Slow-Motion Violin Bow-Control Task: Evidence for the Constrained Action Hypothesis in Bowed String Technique

**DOI:** 10.1177/00224294211034735

**Published:** 2021-08-05

**Authors:** Emma Allingham, Clemens Wöllner

**Affiliations:** 1 University of Hamburg, Hamburg, Germany

**Keywords:** focus of attention, violin performance, motor skill performance, expertise, electromyography

## Abstract

The constrained action hypothesis states that focusing attention on action outcomes rather than body movement improves motor performance. Dexterity of motor control is key to successful music performance, making this a highly relevant topic to music education. We investigated effects of focus of attention (FOA) on motor skill performance and EMG muscle activity in a violin bowing task among experienced and novice upper strings players. Following a pedagogically informed exercise, participants attempted to produce single oscillations of the string at a time under three FOA: internal (on arm movement), external (on sound produced), and somatic (on string resistance). Experienced players’ number of bow slips was significantly reduced under somatic focus relative to internal, although number of successful oscillations was not affected. Triceps electromyographic activity was also significantly lower in somatic compared to internal foci for both expertise groups, consistent with physiological understandings of FOA effects. Participants’ reported thoughts during the experiment provided insight into whether aspects of constrained action may be evident in performers’ conscious thinking. These results provide novel support for the constrained action hypothesis in violin bow control, suggesting a somatic FOA as a promising performance-enhancing strategy for bowed string technique.

Virtuoso music performance exemplifies some of the most impressive feats of motor control of which humans are capable. Musicians spend a huge proportion of their lifetime acquiring these skills (Watson, 2006), and it is the job of music educators to nurture effective skill acquisition and performance habits in their students. Research on motor performance has evidenced that the object of a performer’s focus of attention (FOA) can significantly affect motor skill learning and performance (Wulf, 2013), but despite the relevance of this effect to music education, only a handful of studies have applied this paradigm to music education contexts. Exploring effects of FOA in musical motor tasks is relevant to the field because it investigates the tangible effects that small changes in a music instructor’s words may have on a performing student. Furthermore, this area of research highlights the need for music educators to equip students with not only technical playing skills but also performance psychology skills (Connolly & Williamon, 2004). Thus, potential benefits of FOA to preparation for and execution of music performance deserve further empirical investigation. In the current study, we aimed to test effects of FOA on motor performance in a violin bow-control task.

## Related Literature

### Focus of Attention Research

Many instrumental music teachers may have experienced the phenomenon of *paralysis by analysis* (Ehrlenspiel, 2001), in which thinking consciously about how a motor skill is executed causes difficulties in action execution. For example, when teachers are asked to explain the mechanics of a particular skill, they may find themselves temporarily unable to perform the skill to their usual standards. This effect has been demonstrated in a wealth of motor control studies, mainly in the context of sports, which have shown that motor performance improves when individuals adopt a focus on the environmental effect or goal of their action (i.e., an external focus) compared to a focus on the movement mechanisms of the action (i.e., an internal focus; for a review see Wulf, 2013). With a tennis serve, this could be the difference between thinking about where the ball should bounce (external focus) or thinking about how the arm should swing the racquet (internal focus). With a violin bow stroke, this could be the difference between thinking about the sound produced (external focus) versus thinking about the arm motion (internal focus).

Some studies have challenged the generalizability of these FOA effects. For example, effects may depend on the attentional load of tasks (Sherwood et al., 2020) or may not hold true for novices (Castaneda & Gray, 2007; Perkins-Ceccato et al. 2003). However, the majority of the research literature on this topic shows support for the beneficial effects of an external focus compared to internal focus, particularly when focus conditions are well controlled and definitions of internal and external foci are consistent with previous studies (Singh & Wulf, 2020; Wulf, 2013).

In the FOA paradigm, attentional foci are induced via verbal instructions, and in some cases, differing effects on performance have been shown between instructions varying in only one or two words (Wulf, 2013). In music teaching, instructions and explanations are often given through verbal communication (Schippers, 2006); therefore, the pedagogical value of FOA research in music lies in exploring the psychological and physiological impact that small changes in verbal communication can have on performers’ ability to play well.

In addition to motor performance, this effect has been shown in motor learning processes such that an external focus may improve skill retention and transfer (Becker & Fairbrother, 2019; Song, 2019). Adopting an external FOA also forms a key part of the *optimizing performance through intrinsic motivation and attention for learning* theory of motor learning (Wulf & Lewthwaite, 2016), which also includes methods of enhancing the student’s expectancies for future performances and promoting learner autonomy. Furthermore, motor performance research has found that an internal focus may cause a physiological change in the motor system through increased electromyographic (EMG) muscle activation, indicating decreased efficiency of muscle use (Marchant & Greig, 2017; Neumann, 2019; Neumann & Brown, 2013; Vance et al., 2004). Such an effect in musicians may have implications for efficient use of the body in performance, which could be important in the prevention of overuse injuries.

### Theoretical Underpinnings

The *constrained action hypothesis* (CAH; McNevin et al., 2003; Wulf et al., 2001) provides a theoretical explanation for the FOA effect. CAH posits that focusing on movement mechanisms evokes self-consciousness in the performer and triggers attempts to control automatized motor processes, resulting in performance impairment. In support of this, a recent study has provided neurological evidence that an internal focus promotes conscious motor processing in the form of increased coherence between verbal, analytical, and motor planning brain regions (Law & Wong, 2020). This theory is also closely connected to issues of performance under pressure. For example, the phenomenon of “choking under pressure” is theorized to occur when a performer tries to consciously apply declarative motor knowledge to an action that had become automatized at an implicit level (Beilock & Carr, 2001; Masters & Maxwell, 2008). Awareness and understanding of CAH can greatly benefit music educators in making informed decisions about how use of language in a music lesson may impact a student’s motor control system and when considering how to best support students in coping with performing under pressure.

CAH also reflects understandings of optimal performance in music. For example, Kenny (2011) suggested that music performance anxiety could be avoided by shifting focus away from the self, whereas loss of self-consciousness and task absorption are indicators of flow states, a marker of optimal performance (Csikszentmihalyi, 1990).

CAH is also relevant to pedagogical approaches when applied to the learning process. For example, the CAH seems to encourage implicit learning approaches (e.g., Poolton & Zachry, 2007), in which the student learns motor skills without explicit instructions, or discovery learning techniques are employed (Bakker, 2018; Fowler, 1966; Raab et al., 2009), where the student is encouraged to problem-solve for themselves. An external FOA could be a useful teaching tool in these approaches because attention is placed on the task outcome rather than the process and direct movement instructions are not given.

### Attentional Focus in Music

FOA effects in music performance is an emerging field. In a seminal study, Duke et al. (2011) found that an external FOA on sound improved temporal evenness in skill transfer of a keyboard playing task relative to internal foci on fingers, piano keys, and piano hammers in a sample of 12 nonpianists, although no effect was observed in the four experienced pianist participants. Similarly, Atkins (2017) showed that expert ratings of experienced singers’ performances were improved under an external focus on filling the room with their sound compared to other foci directing their voice to different objects in the room or focusing on their soft palate or vibrato. A study on the expressive performances of a group of various experienced instrumentalists found that an external focus on playing for the audience and the expressive sound of the music produced higher expert performance ratings compared to an internal focus on movement technique and note accuracy (Mornell & Wulf, 2019). These studies offer support for the CAH in musical contexts and suggest the suitability of inducing external FOA as a music pedagogical tool for improving performance.

However, other studies have yielded less clear results. For example, a study on FOA in untrained singers found improvements in expert ratings of performance quality when focusing externally on a point on the wall and on a microphone but also found improvements when adopting an internal focus on feeling the vibrations in the zygomatic arch of their cheekbones (Atkins & Duke, 2013). The authors noted that tactile sensory feedback may have influenced motor behavior in this condition. In addition, Stambaugh (2017) found no significant effects of FOA on skill retention and transfer in a MIDI wind-controller task in a sample of both novice and experienced woodwind players and no effect of FOA on the performances of middle school brass and woodwind players (Stambaugh, 2019).

Current music research on FOA also provides little consensus on the influence of expertise on FOA in music. In sports motor-performance contexts, it has been asserted that the benefits of an external over an internal focus hold true for both experts and beginners (Wulf, 2013), although recently, Singh and Wulf (2020) found that beginners may benefit more from a *proximal* external focus (i.e., closer to the body), which allows them to concentrate on movement technique. This is in opposition to the previously established *distance effect* (Singh & Wulf, 2020), that FOA further from the body are more beneficial (e.g., McNevin et al., 2003). In a music context, the role of expertise in FOA effects requires more attention so that educators may be advised as to how optimal foci may be likely to vary among different levels and abilities.

Researchers have also shown that an internal focus in the context of instrumental music-making might provide beneficial attention to tactile feedback (Stambaugh, 2019), for example, feeling vibrations or tension changes in the instrument. Certainly, the presence of tactile feedback has been shown to be important in consistency of expressive piano performance (Wöllner & Williamon, 2007), but how attention to tactile feedback affects music performance remains largely unstudied. In sports research, it has been theorized that elite athletic performers are likely to cultivate attention to bodily sensations (i.e., tactile and proprioceptive feedback) to maintain spontaneity, skill improvement, and injury avoidance (Shusterman, 2009; Toner et al., 2015; Toner & Moran, 2015). There also exists a parallel to this idea in music education, where somatic training methods such as the Alexander Technique (Cotik, 2019) or Feldenkrais method (Lee, 2018) encourage an awareness of body sensations as a way of learning more efficient use of the body. Indeed, it has been shown that somatic training methods may unwittingly capitalize on external FOA by encouraging attention to movement quality rather than movement mechanics (Mattes, 2016). Furthermore, attention to tactile feedback may have a particular relevance to learning a musical instrument (Stambaugh, 2019). Attention to tactile feedback through the instrument might, for example, play a role in developing a feeling for the instrument as an extension of the performer’s body (Nijs, 2017). From a pedagogical perspective, deepening understandings of how attention to tactile feedback affects the motor system can inform instrumental teaching approaches. Thus, it is useful to investigate how a focus on tactile sensory feedback (i.e., a proximal external focus) through a musical instrument would compare with more standard internal and external foci.

### FOA in Violin Bow Control

A fundamental tenet of string pedagogy is cultivating the ability to produce a beautiful sound quality (Galamian, 1962), a skill that can take years to master (Konczak et al., 2009). Central to this tone production skill is learning to balance bow speed, pressure, and contact point (i.e., position on the string) to produce the high amplitude harmonics (i.e., resonant frequencies of the fundamental tone) characteristic of good quality string sound (Collins, 2009). Producing this type of resonant string vibration has been described in terms of the physical string motion, in which the string first sticks to the bow and is pulled to one side, before slipping back to its original position (i.e., Helmholtz motion; Schoonderwaldt, 2009). The renowned pedagogue Simon Fischer created a useful slow-motion bowing exercise for developing a feeling for this stick-slip pattern and thus cultivating tactile sensitivity to the amount of downward bow force required for strong tone production (Fischer, 1997). The exercise involves a slow-motion version of the slip-stick pattern in which the student attempts to create single oscillations of the string at a time (see “The Current Study” section for more detailed description). This bowing exercise is a valuable pedagogical strategy because it distills the motor-control skills needed for good tone production into a slow, thoughtful task with clear performance feedback. Thus, the question of how FOA might impact bow-control ability during this slow-motion bowing task is highly relevant to pedagogical approaches of teaching tone production.

In a previous study in a violin tone-production task, we found that an FOA on tactile feedback increased tone brightness, reduced shoulder muscle activity, and increased novices’ violin sway relative to an arm movement focus while improving consistency of bow-bridge distance relative to a sound focus (Allingham et al., 2021, in review). These results suggest benefits of a focus on tactile feedback in violin open-string tone production. With the current study, we aim to extend these findings to the highly nuanced bow-control skills of this slow-motion bowing exercise.

## The Current Study

We investigated effects of attentional focus instructions on motor-skill performance in a violin-bowing task in both expert upper strings instrumentalists and bowed string instrument novices. We used a pedagogical slow-motion bow exercise, which aims to train nuanced bow-control skills necessary for producing good violin tone (see the following for the task description). The exercise requires a “less is more” approach, allowing the string to “do the work for itself,” making it particularly suitable for exploring constrained action. Finally, the task enabled us to test CAH in the novel context of a very slow movement with an unfamiliar exercise to both experienced and novice players. As well as the standard internal and distal external foci, we included a novel focus intended to bring awareness to tactile feedback through the bow. We termed this a *somatic focus* because our aim was to mimic the kind of external FOA prompted by somatic training methods. This focus is, by Wulf’s (2013) definition, an external focus because it does not refer directly to body movement; however, it is also intended to bring attention to sensations at the border of the internal-external dichotomy (Stambaugh, 2017) and thus may be viewed as being in between internal and external. Such a focus on tactile sensations may be positioned as either an internal or external focus depending on the instructions given, and in the current study, because the instructions refer to the instrument rather than the performer’s body, the focus on tactile feedback constitutes an external focus. In addition to measuring FOA effects on task performance (see the following for details), we also explored physiological effects by measuring muscle activity in the bowing arm. This was informed by findings in sport that an internal FOA promotes inefficiency of muscle use (e.g., Neumann, 2019; Vance et al., 2004).

We hypothesized that the two external foci (on sound and on bow-string resistance) would result in fewer errors and more successful sounds in the bow-control task as well as reduced muscle activity (i.e., indicative of increased motor efficiency) compared to the internal focus (on arm movement). We also investigated whether experienced and complete novice string players would respond differently to FOA. Additionally, we aimed to explore how constrained action effects might be reflected in performers’ conscious thoughts via an exploratory text-based analysis of participants’ reported thoughts during the bowing task.

## Method

### Participants

We recruited 33 participants, 18 female and 15 male, between the ages of 19 and 42 (*M* = 24.97 years, *SD* = 4.80), all of whom were right-handed, via mailing lists and online advertising. We aimed to have a group of experienced violin/viola players (at least 10 years of training) and a group of novices (no experience playing a string instrument). We chose to recruit novice string players with training in another musical instrument to control for overall musical expertise and to ensure the training process was not too difficult for novice participants. We included viola players in the experienced group because the motor skills required for bowing on violin and viola are very similar. All participants performed the experimental task on a violin. We later excluded one participant from analysis when it became clear that this participant’s level of violin training was notably lower than the experienced group but too high to qualify for the novice group. This resulted in a sample of 32 participants, 18 female and 14 male, between the ages of 19 and 42 (*M* = 24.94 years, *SD* = 4.87), all of whom played a musical instrument. The novice group had 16 participants between the ages of 21 and 37 (*M* = 24.56 years, SD = 3.88) who had studied an instrument that was not violin or viola for 2 to 21 years (*M* = 11.84 years played, *SD* = 4.66). The experienced group had 16 participants between the ages of 19 and 42 (*M* = 25.31 years, *SD* = 5.80) who had studied violin or viola for 10 to 36 years (*M* = 18.56 years played, *SD* = 6.12).

### Equipment and Experimental Setup

We carried out the experiment in a 5 m × 5 m laboratory. All participants used the same violin, which was a Fastoso intermediate student model, mounted with an AKG Harman C411PP contact microphone. The microphone recorded sound through Audio Desk software and a MOTU 828MK3 audio interface. A green sticker was placed on the stick of the bow to mark the starting point for the bowing task. Trigno Delsys wireless surface EMG sensors were placed on the participant’s bicep, triceps, and deltoid (i.e., shoulder) of the right arm. EMG data were recorded through Qualisys software and synchronized with audio via SMPTE timecode.

### Procedure

Participants provided written informed consent before taking part in the study in accordance with the Local Ethics Committee guidelines. They answered a short demographic questionnaire and several questions about their musical training history. Next, EMG sensors were fitted in line with Surface ElectroMyoGraphy for the Non-Invasive Assessment of Muscles guidelines (see www.seniam.org), and the signal to noise ratio was visually inspected to ensure correct placement. Because the current data collection took place alongside another study in which motion-capture data were collected, participants were also outfitted with Qualisys motion-capture jackets and reflective markers. Although the details of the motion-capture collection are not relevant to the current study, we wish to point out that this extra equipment was present during data collection. The motion-capture equipment was flexible, lightweight, and designed to allow a wide range of motion; therefore, it did not restrict movement or impede performance in the current study. The experimenter took care to ensure that the jackets did not disturb the EMG sensors through careful checking of sensor placement and inspecting the EMG signal. The first author (an experienced violin teacher) then carried out a short training session, teaching participants the bowing task and explaining the experimental procedure. For novices, the training lasted about 15 to 20 minutes and included learning the basics of holding the violin and bow. For experienced violinists, the training lasted about 5 to 10 minutes. During the training, the first author established that participants were able to cope with the basic technique, able to generate the desired *click* sound, and able to recognize a correct click compared to an error (i.e., a *bow slip*; see the following for details). Because Wulf (2013) pointed out that visual gaze should be controlled across focus conditions to avoid confounds, participants were instructed to keep their gaze on the violin A string during the task and not to look around the room while performing.

We carried out the experimental procedure alongside a second study comprising the same participants and the same focus instructions but different bowing tasks and separate analyses (Allingham et al., 2021, in review). We devised the two studies a priori to be analyzed separately, with different research questions and different dependent variables but one long, single data-collection session per participant. For each focus condition, participants carried out Bowing Task 1 (not analyzed in the current article), followed by Bowing Task 2 (detailed in the following). Task 1 comprised a simple open string bowing exercise and thus also provided a warm-up for the second task. In between each condition, participants were asked to verbally report what they had been thinking about during the tasks, and these comments were recorded on audio and transcribed by the researcher. Participants then sat quietly for 1 minute before the next condition to minimize carryover effects.

The bowing task is taken from Simon Fischer’s (1997) book *Basics: 300 Exercises and Practice Routines for the Violin.* The exercise is a slow-motion sound production task where the student attempts to create single oscillations of the string at a time, each of which result in a small click sound. This click is the result of pulling the string to its maximum stretching point, releasing, and catching it again before it can continue to vibrate. If the performer pulls too hard, the bow will slip, creating a scratchy sound, and if they don’t pull hard enough, they will produce no sound. As the bow travels, the performer encounters different parts of the bow, each with varying tension levels in the hair, and so the precise amount of downward bow force and lateral pull required to produce an oscillation varies with each attempt. Thus, the task requires very nuanced, slow motor-control skills, which underpin the fundamentals of quality sound production in string playing. The task allowed us to have a clearly quantifiable performance success metric (number of clicks and number of errors) while still being an ecologically valid bow-control task with direct relevance to the teaching of sound-production skills.

For each trial, participants were given 30 seconds to carry out the task, with the goal of making as many clicks and as few mistakes as possible. The exercise was carried out in a down bow direction, starting near the heel, and participants were instructed not to lift the bow off the string or change direction. They performed three trials for each focus condition. Participants were told they would be given instructions on what to think about during the task, and the focus instructions were then given verbally and reinforced for every trial. The focus instructions were:

**Internal:** Focus your attention on the movement in your right arm.
**External:** Focus your attention on the sound you produce.
**Somatic:** Focus your attention on the resistance of the bow against the string.

### Data Analysis

#### Manipulation check

The reported thoughts given after each focus condition were analyzed to establish how well participants were able to follow the focus instructions. The first author coded each comment as either evidencing that the instruction was followed or not. Overall, 93% of all comments were judged as having followed the focus instructions, and no single participant was judged as unable to follow the instruction in all three conditions, indicating a high success rate.

#### Audio

The first author manually scored each audio recording by counting the number of correct clicks and errors (i.e., bow slips) using both audio and visual inspection of the sound wave and averaged scores across trials.

#### Electromyography

The EMG sensors contained an initial band-pass filter of 20Hz to 450Hz. All further processing was carried out using custom software in MATLAB. Data were first visually inspected, and one bicep muscle trial was excluded from the analysis due to movement artifacts. This exclusion comprised 0.005% of total EMG data points. Data were then mean centered and full-wave rectified, and a moving root mean squared (RMS) filter was applied (50-ms window length, 25-ms overlap) to give a measure of the power of the signal. This RMS curve was then maximum–minimum normalized to control for individual differences, and the mean RMS value (millivolts [mV]) was derived to indicate the average power of muscle activity during each trial. These values were averaged across trials.

#### Reported thoughts while performing

We carried out an exploratory text-based analysis of participants’ reported thoughts during the experiment (using the same data used for the manipulation check), with the aim of exploring whether indications of constrained action effects might be evident in participants’ recollected conscious thoughts. The data consisted of one comment per focus condition, in which the participants described what they had been thinking about (96 comments in total). To summarize the comments, five themes were derived from the data by the first author with a view to constrained action theory and somatic pedagogy. First, the theme of *curiosity* (displaying curiosity, interest in the process and exploring technical issues) aimed to capture evidence that participants were absorbed in the task itself rather than feeling self-conscious, in line with the notion that constrained action arises from a self-invoking trigger (Wulf & Lewthwaite, 2010). In opposition, the theme *trying hard* (feeling anxious about one’s own performance or aiming for perfection) aimed to capture preoccupation with the self or self-performance. The *letting go* theme (relaxing, not caring about mistakes) aimed to indicate instances where participants felt relaxed and unconcerned about their performance, which would indicate absence of the conscious control brought about by constrained action (Wulf et al., 2001). The theme *noticing sensations* was conceived in line with somatic training pedagogy, aiming to indicate moments of nonjudgmental somatic awareness (Lee, 2018), and the theme of *physical discomfort* captured the experience of more negative body sensations. Thus, the presence of the trying hard theme might indicate constrained action effects, whereas curiosity, letting go, and noticing sensations themes may indicate the absence of such an effect. The physical discomfort theme may or may not relate to constrained action effects but nonetheless captured a relevant aspect of the data.

Comments were then randomized so that the focus condition/participant to which each comment belonged was obscured. The first author and a second coder then coded each comment with a 0 (theme not present) or 1 (theme present) for each theme in nonexclusive categories. This process was implemented to develop a single variable, reducing coder bias. Because Cohen’s κ between the first two coders yielded an interrater agreement, κ > .5 (*M* = .55), for all themes, indicating reasonable agreement, a third independent coder was brought in to resolve disputes and produce final dichotomous variables with 100% agreement. Therefore, where there was disagreement, the majority decision of the three coders was taken.

#### Statistical analyses

Statistical analyses were carried out in R Project for Statistical Computing Software, Version 4.0.2 (www.r-project.org). All variables were screened for outliers such that values greater or less than 3 *SD* from the mean were excluded on a case-wise basis. This resulted in the removal of two outliers from the errors variable, one from clicks and one from the deltoid EMG variable. For the five quantitative dependent variables (number of clicks, number of errors, and EMG activity of bicep, triceps, and deltoid), we carried out individual mixed analyses of variance with focus condition (3) as the repeated measures factor and expertise (2) as the between-groups factor. Where Mauchley’s test of sphericity was significant, we report the Greenhouse-Geisser corrected degrees of freedom. We report Bonferroni-corrected post hoc pairwise comparisons for any statistically significant main effects, and for any statistically significant interactions, we report simple effects analysis, also with Bonferroni-corrected *p* values.

## Results

### Audio

**Figure 1.**
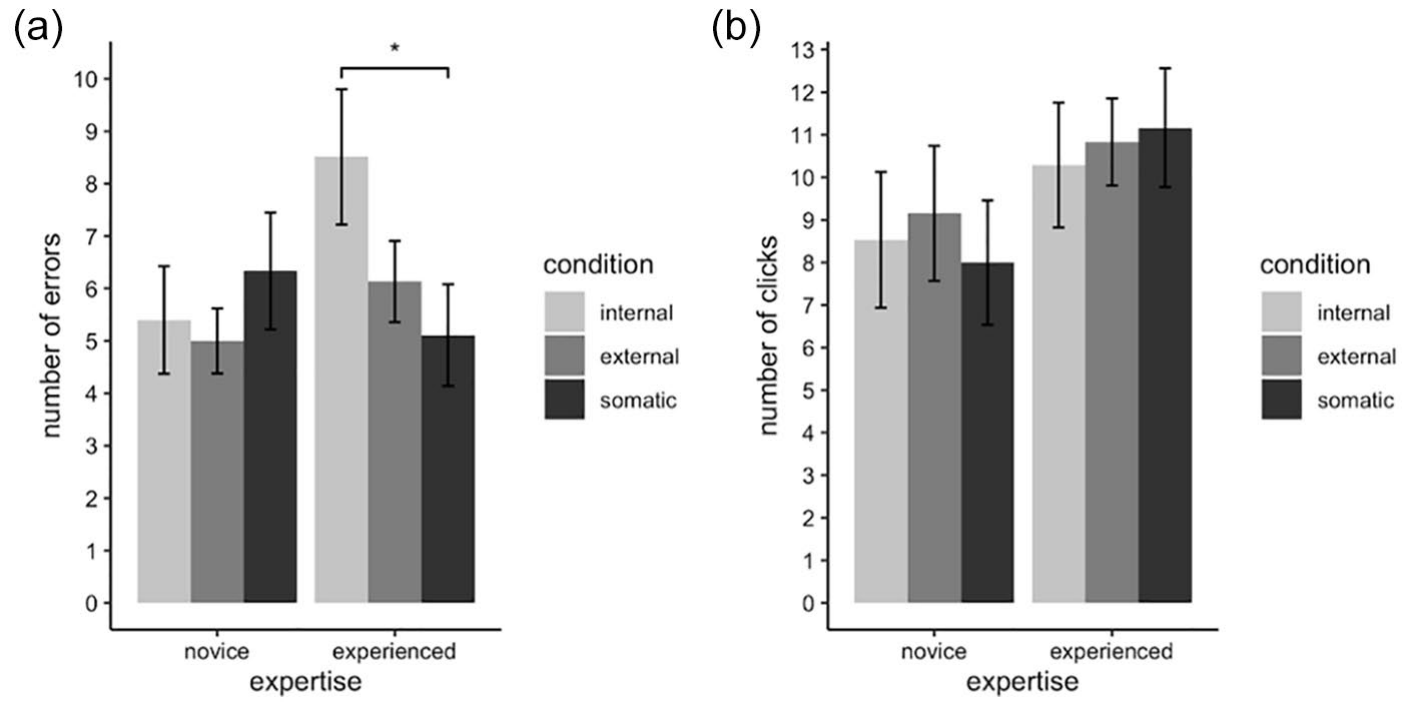
Audio results. *Note.* Bar graphs depicting (a) mean number of errors and (b) mean number of clicks for each focus condition within each expertise groups. Error bars indicate the standard error of the mean. *p < .05.

We analyzed outcome variables number of errors and number of successful clicks to investigate effects on task performance. For number of errors, there was no main effect of focus condition, *F*(2, 56) = 2.37, *p* = .102, η_*p*_^2^ = .08 (see Figure 1a), and no effect of expertise, *F*(1, 28) = 0.77, *p* = .387, η_*p*_^2^ = .03; but there was a significant interaction effect, *F*(2, 56) = 4.83, *p* = .012, η_*p*_^2^ = .15. A simple effects analysis revealed a statistically significant effect of focus condition for experts, *F*(2, 28) = 6.36, *p* = .01, η_*p*_^2^ = .31, but no significant effect for novices, *F*(2, 28) = 0.94, *p* = .80, η_*p*_^2^ = .06. Pairwise comparisons for the expert group revealed significantly higher number of errors in internal (*M* = 8.51, *SD* = 5.00) compared to somatic (*M* = 5.11, *SD* = 3.76, *p* = .016), with no significant differences compared to external (*M* = 6.13, *SD* = 3.00). This result indicates that experts made significantly more errors when focusing on arm movement compared to focusing on string resistance.

For number of clicks, there was no main effect of condition, *F*(2, 58) = 0.21, *p* = .81, η_*p*_^2^ = .007; no effect of expertise, *F*(1, 29) = 1.65, *p* = .21, η_*p*_^2^ = .05; and no interaction effect, (see Figure 1b). There was no evidence in either variable that expertise had a main influence on task performance.

### Electromyography

**Figure 2.**
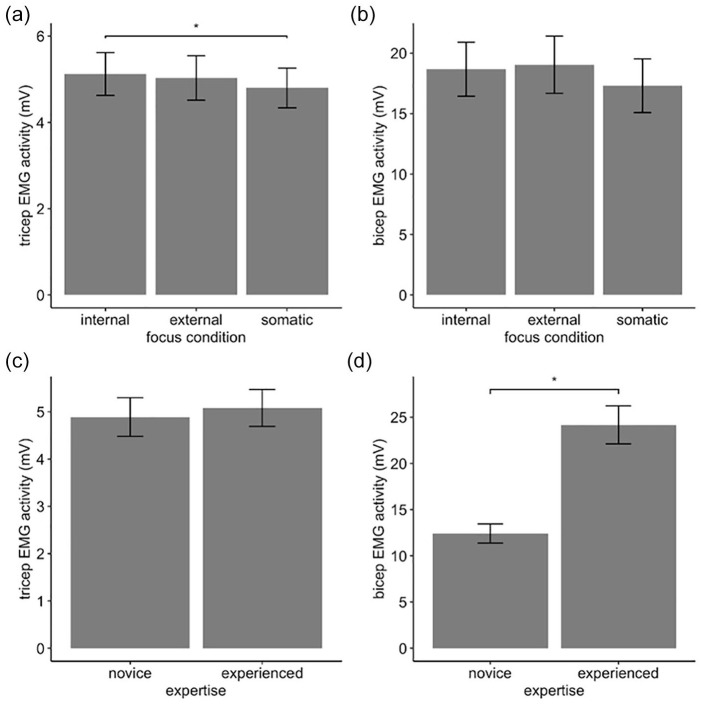
Electromyographic results. *Note.* (a) Significant main effect of focus condition on triceps muscle, (b) nonsignificant effect of focus condition on bicep muscle, (c) nonsignificant effect of expertise on triceps, and (d) significant main effect of expertise on bicep muscle activity. Error bars indicate standard error of the means. *p < .05.

We analyzed EMG muscle activity to determine physiological effects of attentional focus. For the triceps muscle, there was a main effect of focus condition, *F*(2, 60) = 3.82, *p* = .028, η_*p*_^2^ = .11 (see Figure 2a), such that the internal focus produced significantly higher muscle activity (*M* = 5.12 mV, *SD* = 2.81) compared to somatic (*M* = 4.80 mV, *SD* = 2.60; *p* = .043), with no significant difference to external (*M* = 5.03 mV, *SD* = 2.92). There was no effect of expertise, *F*(1, 30) = 0.04, *p* = .85, η_p_^2^ = .001, and no interaction effect.

For the bicep, there was no main effect of focus condition, *F*(1.54, 44.8) = 3.09, *p* = .07, η_*p*_^2^ = .10, but there was a main effect of expertise, *F*(1, 29) = 8.78, *p* = .006, η_*p*_^2^ = .23 (see Figure 2b), such that novices had significantly lower muscle activity (*M* = 12.42 mV, *SD* = 7.11) than experienced players (*M* = 24.17 mv, *SD* = 14.26). There was no interaction effect.

For the deltoid muscle, there was no significant effect of focus condition, *F*(2, 58) = 1.78, *p* = .18, η_*p*_^2^ = .06, or expertise, *F*(1, 29) = 0.16, *p* = .69, η_*p*_^2^ = .006, and no interaction effect.

### Participants’ Reported Thoughts

**Figure 3.**
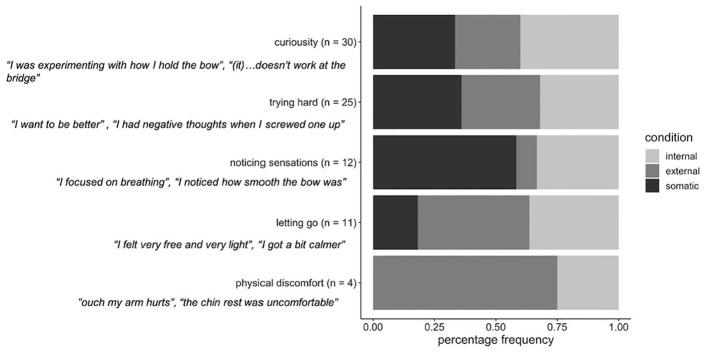
Summary of participants’ reported thoughts. *Note.* Distribution of focus conditions within comments relating to each theme. The *y*-axis displays each theme derived, with the total number of comments coded as belonging in each theme in brackets, along with two example comments. The *x*-axis displays the frequency, as a percentage, of each focus condition within each theme.

Figure 3 displays the percentage distribution of participants’ comments for each theme across the focus conditions. From these descriptive trends, we can observe that the curious and trying themes are fairly evenly distributed across all three focus conditions. This is a little surprising because we might have expected the internal condition to reflect more frequent trying and less frequent curious comments, in reflection of CAH. On the other hand, the letting go theme appeared mostly in the external and somatic foci, and the noticing sensations theme appeared mostly in the somatic focus, which might indicate changes in thought content related to the focus induced. Because the prevalence of some themes was very low, statistical analysis of these differences was not possible in the current sample.

Some additional insights were noted in these data that can inform further research. For example, one experienced player had the opinion that focus strategies could/should be combined for an optimal performance: “concentrating on the sound combines the two tasks before (i.e., focusing on movement, and string resistance) . . . [you] have to be aware of both to get a good sound.” This comment highlights the importance of considering the ecological validity of focus instructions. In addition, some participants seemed to show an intuitive understanding of constrained action effects, realizing that they had to yield control and declarative knowledge to succeed at the task. For example participants reported: “I had to let go a bit and it worked better than concentrating too hard” and “I find that focusing hard on movement in the arm has the opposite effect of what it should. The arm stiffens up: it’s counter-productive.” These unprompted reflections on the detrimental effects of the internal focus align with CAH.

## Discussion

The results of this study provide partial support for the CAH in the novel context of a slow-motion bow-control task and suggest benefits to bow control of a somatic FOA (a type of external focus) on tactile sensory feedback. We found that the somatic focus improved task performance for experienced string players but not for novices, suggesting that expertise was an influential factor. We also observed that the somatic focus decreased triceps muscle activity compared to internal for both groups, consistent with physiological understandings of constrained action. These findings tentatively suggest that encouraging attention toward tactile feedback through external focus instructions instead of internal movement mechanisms may more optimally support bow-control performance skills, particularly with experienced performers. However, there were no significant differences between the internal focus on arm movement and the external focus on sound, which does not support previous findings that focusing on sound improved music performance (Duke et al., 2011). We also observed no significant effect of attentional focus on number of successful click sounds or on muscle activation in the bicep or deltoid muscles, which was contrary to our hypothesis. Participants’ reported thoughts during the experiment provided insight into how aspects of constrained action may or may not be evident in performers’ conscious experience.

### Task Performance

In partial support of our hypothesis, we found that experienced players made significantly fewer errors under the somatic focus on bow-string resistance compared to the internal focus on arm movement. This result is in line with previous research supporting the CAH in music tasks (Atkins, 2017; Duke et al., 2011; Mornell & Wulf, 2019) and suggests that the bow-control skills essential for quality violin tone production are better supported by a focus on bow-string resistance than a focus on arm movement. However, we found no significant difference in errors between the internal and external foci, suggesting that for this task, a focus on sound was not a beneficial alternative to an internal focus. This finding is in line with our results from a previous study on FOA in a different task of open string bowing (Allingham et al., 2021, in review) in which we similarly found that a somatic focus increased spectral centroid of violin tone and reduced shoulder muscle activity compared to internal focus but that there were no significant differences between the internal and distal external foci.

This result could be interpreted as opposing the *distance effect*, which would have predicted better task performance in the external focus on sound compared to the arguably more proximal somatic focus. This could be an effect specific to violin playing, supporting the notion that attention to tactile sensory feedback can be especially beneficial in instrumental music-making (Stambaugh, 2019), particularly string playing. Furthermore, this finding may be driven by a similar mechanism to that of Singh and Wulf (2020), who found that for beginner volleyball players, a proximal external focus, which brought attention to the technical means of action execution, was more effective than a distal external focus. In our study, the somatic focus may have allowed performers to concentrate on bow technique while avoiding constrained action effects, whereas the sound focus did not allow this attention to technique and therefore was not helpful. Although Singh and Wulf found this effect only for beginners and not experts, our study used a task that was unfamiliar to both expertise groups, meaning that experienced players may have been responding more like beginners. However, it should be noted that a focus on sound might not necessarily constitute a distal focus given that sound might be experienced as close to the body. Further research is needed to establish whether performers experience a focus on sound as distal or proximal. Furthermore, the lack of FOA effect in our novice group may have been due to poorly established bow technique, wherein a suboptimal bow hold may have made the current task easier, giving the novices an advantage. This explanation would also be in line with our findings that experienced players did not perform better overall than novices.

Another possible explanation of the observed expertise effects is that novices might have felt less pressure to perform well than the experienced players, making them less susceptible to constrained action effects. This would be in line with Gray (2004), who found that experts were more likely than beginners to reinvest procedural knowledge into a task when they were performing poorly in an attempt to learn what they were doing wrong and regain control over task execution. Under this reasoning, the poor performance observed in the experienced group may have been a longer term strategy to learn from their mistakes. Indeed, this explanation would be consistent with our result that experts had overall higher bicep activity than novices, possibly reflecting increases in small elbow flexion movements in attempts to regain control after errors. Further research using the current task in a skill-retention test could investigate whether experts are likely to exhibit similar immediate performance but better skill retention than novices.

Considering in more detail the apparent inefficacy of the external focus in this particular task, we propose a possible explanation in the immediacy of the different feedback sources focused on. For example, focusing on bow-string resistance drew participants’ awareness to in-the-moment feedback about string behavior, even possibly highlighting cues about what the string would do next. In contrast, focusing on sound placed participants’ attention on more delayed feedback. The increased immediacy of the tactile feedback to which performers attended in the somatic focus may have increased their ability to react quickly enough to avoid errors. This highlights the potential usefulness of a focus on bow-string resistance for supporting technical bowing precision. However, a focus on sound may be more effective in different types of musical tasks, particularly more highly automated tasks.

Another aspect that should be considered is how our external instruction to focus on the sound produced might perform in comparison to a focus on imagined sound. Wulf (2016) argued that FOA instructions given in a sports context affect the preparation stage of action execution, suggesting the idea that an external focus might elicit preparatory visual imagery. In music, a more equivalent type of external focus might be to focus on imagined sound rather than actual sound. Because auditory imagery is widely used in mental rehearsal strategies among musicians (Connolly & Williamon, 2004), it would be interesting to explore how use of auditory imagery may be combined with FOA techniques to optimize music performance. Although our results support the benefits of a somatic FOA over an internal FOA in this specific bow-control task, they also highlight that various types of external focus may have differing effects and that attention to certain kinds of feedback may affect performance. This supports previous findings in music research that a distal external focus does not always improve performance and learning (Atkins & Duke, 2013; Stambaugh, 2017, 2019).

Additionally, we wish to highlight the novelty of these findings in the context of a very slow movement. Because slow movement may cause changes in attentional state (Niksirat et al., 2017), it is possible that the slowness of the task is responsible for the observed benefits of the somatic focus compared to the external one. Further studies on this topic should evaluate FOA effects at differing movement speeds to explore the potential influence of slowness.

Our hypothesis that FOA would increase number of clicks that participants were able to produce was not supported. One explanation for this could be that conscious control elicited by the internal focus caused participants to increase their rate of attempts along with decreasing bow-control ability, resulting in an increase in task success along with the number of errors. Further research could explore how FOA affects pace of behavior or perception of passing time. Nonetheless, our overall results on task performance indicate benefits to bow-control performance of focusing on tactile feedback.

### EMG Activity

In line with previous research on the physiological effects of an internal focus, we found significantly increased muscle activity under internal focus compared to somatic for the triceps muscle. However, contrary to our hypothesis, we observed no effect of FOA in the bicep or deltoid muscle. The specificity of this effect to the triceps muscle is presumably due to the nature of the task, which involved only down-bow actions (i.e., triceps muscle use). In sports-based motor-performance research, such differences in EMG activity have been attributed to changes in muscle efficiency. However, in our study, it is difficult to tell whether this result reflects muscle efficiency because we did not control number of movements between conditions. Further research could aim to test efficiency of muscle use in music performance under different FOA by controlling for number of repetitions of a specific movement. Nonetheless, our findings are partially consistent with previous literature that an internal focus causes increased muscle activity relative to external. This finding cautiously supports the notion that a music teacher’s verbal instructions drawing attention to internal movement mechanisms may not optimally support efficient muscle use in students. Another useful question for further research would be the relationship between FOA and overuse injuries in musicians. For example, if an external focus causes more efficient muscle use in musicians, FOA might be a useful pedagogical tool for avoiding injuries caused by excess tension.

### Participants’ Reported Thoughts

Our exploratory analysis of participants’ reported thoughts during the experiment provides an initial overview of how FOA might or might not be reflected in performers’ conscious thoughts. For example, the letting go theme showed a trend for appearing less often in the internal focus, in line with ideas of constrained action as increased conscious control (Wulf et al., 2001), but the trying hard and curiosity themes did not seem to differ across focus conditions. Further research could look for systematic effects of attentional focus in performers’ reported thoughts. It is also interesting that the physical discomfort theme did not appear in the somatic focus condition at all, and further research could explore whether a somatic focus might reduce thoughts about negative physical sensations. In addition, some participants reported a preference to focus on several objects at once, indicating that for some individuals, focusing on one aspect of performance at a time may not be an ecologically valid approach. Finally, some participants communicated an intuitive understanding of the detrimental effects of an internal focus, providing support for the phenomenon of constrained action from an experiential point of view.

### Limitations

The generalizability of our findings is limited by the specificity of our sample, which consisted of mainly German, undergraduate, amateur musicians, all of whom specialized in classical music. Further research should aim to sample musicians of various ages, stages, musical genres, and nationalities. Studies of FOA effects in children are particularly relevant to music education research. Also important to music education would be investigating how individual differences, such as personality traits and experience with somatic training methods or mindfulness, may influence FOA effects. For example, training in the ability to observe the body in a nonjudgmental, noncontrolling way might limit the detrimental effects of an internal focus (Mattes, 2016). Our findings are also limited to the particular task we chose to study, which was a reductive technical exercise (albeit, a pedagogically relevant task) rather than a fully expressive musical performance. Only one rater carried out the audio analysis, which may affect the generalizability of these findings. However, the assessment of task performance through both audio and visualization of the signal improved the objectivity of this measure. A next important step will be to understand the meaningful impact this effect might have on real-life violin performance skills. Expressive music performance requires a distinct set of sensorimotor and cognitive skills in comparison to technical exercises (Kenny, 2011) and thus may necessitate a different optimal FOA. An abundance of musical tasks remains to be investigated in this context. In particular, FOA effects on intonation skills in string playing, layperson perceptions of expressive performance, and changes to expressive gesture could be particularly interesting. There is also still a need to extend research on FOA effects to the learning of musical motor skills as well as performance (Stambaugh, 2017, 2019) and to apply the FOA paradigm to real music performance situations outside the laboratory and to group music-making contexts.

### Pedagogical Implications

The results of our study suggest that focusing on internal movement mechanisms may be detrimental to the performance of a violin bowing motor task, whereas a focus on tactile sensations through the bow may be beneficial. The bow-control task used in the current study is based in a realistic educational exercise that condenses the motor skills necessary for high-quality violin sound production; therefore, our findings are directly relevant to violin teaching approaches. We suggest that promoting a somatic FOA during performance should have a good potential for improving violin bow-control skills. Although further research is needed to test FOA effects in a more applied educational context, the findings presented here can inform and inspire educators to consider the effects that FOA may have in their day-to-day teaching. Trying out different attentional foci in teaching practice is a safe, easy, and potentially powerful educational tool. Building on recent initiatives from music conservatoires to incorporate mental skills and health and well-being training into their curricula (Connolly & Williamon, 2004; Matei et al., 2018), FOA offers a performance psychology technique that can be incorporated easily into everyday lessons and practice. This provides a practical contribution toward the need to equip music students with evidence-based performance skills (Ford, 2013; Shaw et al., 2020). Furthermore, in a learning context, this kind of technique-based external focus could be a useful educational tool to promote implicit or discovery learning (Bakker, 2018; Poolton & Zachry, 2007). For example, by focusing on tactile sensations, the student is encouraged to learn the rules of bow control through interaction with the instrument and without the need for explicit instructions on bow technique. In this way, a somatic focus might also allow teachers to guide students toward understanding the association between physical action and acoustical outcome (Parsons & Simmons, 2020) while avoiding constrained action effects. Indeed, FOA remains a promising music-performance-enhancing technique, deserving the attention of today’s music educators.
